# Dr. Green’s Case of Amputation of the Leg, with Some Observations on a New Mode of Amputating

**Published:** 1842-09-24

**Authors:** Thomas Green

**Affiliations:** Surgeon to St. Peter’s Hospital, and Lecturer on Surgery at the School of Medicine, Bristol


*Case of Amputation of the Leg, with some Observations on a New Mode of Amputating.* By THOMAS GREEN, Surgeon to St. Peter's Hospital, and Lecturer on Surgery at the School of Medicine, Bristol.—James Allpass, aged thirty-four, a butcher, was admitted into St. Peter's Hospital, on Feb. 22, 1842, under the care of Mr. Green.

About four years ago the patient met with an accident, which caused a severe compound fracture of the right leg. After some time the bone united, not in a straight line, but obliquely, and there is now a considerable projection of bone at the seat of the fracture, around which the skin has been extensively ulcerated for some time. He has been repeatedly in the hospital; on each occasion the state of the ulceration was improved; but immediately on attempting to walk, it again degenerated into a state of foul extensive ulceration. On admission, the ulcer was found in a sloughing condition, with copious sanious discharge; the surrounding skin was of a fiery red colour, and the whole extremely painful; the limb is shortened, and he cannot bring the heel to the ground; tougue coated; pulse quick; complains of cough and loss of rest.

Finding that the leg was entirely useless, and that he had suffered in general health from the last extension of ulceration, he consented to the removal of the limb; but the operation was deferred until he was in a fit state to undergo it.

March 6th.—The cough has now ceased; tongue clean; pulse natural; he sleeps well; the ulceration is in an improved condition, and the skin healthy below the knee.

Mr. Green decided on performing the flap amputation, but in a different manner from that in which it is usually done. A transverse incision having been made across the front of the leg, through the skin, another was made through the integument at the back of the limb, including a large portion of the calf, and leaving skin enough to cover the flap of muscle, which was next formed by passing the catlin through the leg a short distance behind the bones, and cutting out in the usual way; the remaining muscles were divided by a transverse incision passing between the bones, which were next sawn through, and the arteries tied. Hæmorrhage still continued from a large vein, which it became necessary to secure by ligature. Three sutures and pieces of strapping were applied to keep the posterior flap in apposition. Cold cloths to be constantly applied to the stump, and to take immediately half a grain of acetate of morphia in a draught.

9th.—The patient has had no unfavourable symptoms whatever until this morning, when hæmorrhage came on from the stump, from which he lost a considerable quantity of blood; the tourniquet was immediately placed round the thigh, and secured, by which the bleeding was arrested. He states that the limb has started occasionally during the night. His system shows the effects of loss of blood; his face and lips are pallid; feels faint and cold; pulse quick and small; tongue pale and tremulous. Cold water dressings to be constantly applied to the stump; to take at once half a grain of acetate of morphia; the tourniquet to be kept loosely over the femoral artery, and tightened immediately on the appearance of fresh hæmorrhage.

12.—There has been no further bleeding until this morning, when the stump bled again to some extent in a few minutes; the tourniquet was immediately tightened, and the hæmorrhage was stopped. Mr. Green having been sent for, directed the same means to be used; the man to be kept perfectly quiet, and closely watched. He stated that, on the return of any fresh hæmorrhage, he would apply a ligature round the femoral artery, considering it useless in the present stage of the wound to attempt to secure the bleeding vessel by opening the stump.

17th.—There has been no loss of blood since last report; a considerable portion of the wound on the fibular side has united.

April 6th.—The wound has entirely healed, except where one ligature hangs out; the others have come away. His general appearance is much improved.

20th.—The ligature only came away to-day, repeated gentle attempts having failed to bring it away previously; stump healed, and in good condition.

On the method of operating resorted to in this case, Mr. Green made the following remarks at the next surgical lecture at the Medical School:—

The mode of operating employed in this case I first tried a few months ago at St. Peter's Hospital, on a man named Morris, and found that by it all the inconveniences alluded to were avoided, for the man left the house with an exceedingly good stump, and now makes good use of his vocation as a sweeper of one of the crossings at Clifton. I shall now describe the operation as I performed it yesterday, and if you examine the limb removed, which lies on the table, you will easily understand its stages. An incision was made anteriorly across the fore past of the leg, in the usual situation, about two inches below the tuberosity of the tibia; it extended from the inner angle of that bone to a point behind the fibular; from the termination of this incision, on the inner side of the leg, the knife was carried downwards, to some extent, next across the limb posteriorily in a curved line, and brought up at the outer side, so as to unite with the front incision behind the fibula. In this manner a portion of the integument was divided, which might be correctly described as representing two-thirds of an oval figure. This incision should go through the skin and subjacent tissue, down to the fascia covering the muscles; the contraction of the integument itself, with a trifling assistance, by drawing the skin upwards, leaving a separation of about half an inch between the edges of the incision. A long catlin was now pushed through the leg, about one-third of an inch behind the bones, and carried downwards and next backwards, so as to make a flap of muscle, its edges corresponding with those of the retracted integument. The remaining muscles were next divided transversely; in this division are contained the large vessels and nerves, which are those cut transversely. The bones were separated, the sharp angle of the tibia was sawn off, and the arteries secured in the usual way. The flap, when brought up over the face of the stump, was entirely and abundantly covered by skin; three sutures were used, assisted by two broad pieces of strapping; a cloth wetted in cold water was applied over the stump, and the man removed.—*Lond. Med. Gaz.*, *from Prov. Med. Journ.*

